# Butrepyrazinone, a New Pyrazinone with an Unusual Methylation Pattern from a Ghanaian *Verrucosispora* sp. K51G

**DOI:** 10.3390/md12105197

**Published:** 2014-10-16

**Authors:** Kwaku Kyeremeh, Kojo Sekyi Acquah, Mustafa Camas, Jioji Tabudravu, Wael Houssen, Hai Deng, Marcel Jaspars

**Affiliations:** 1 Marine and Plant Laboratory of Ghana, Department of Chemistry, University of Ghana, Accra, P.O. Box LG 56, Ghana; E-Mail: ksaquah@yahoo.com; 2 Department of Bioengineering, Faculty of Engineering, Tunceli University, 62000 Tunceli, Turkey; E-Mail: mustafacamas@gmail.com; 3 Marine Biodiscovery Centre, Department of Chemistry, University of Aberdeen, Old Aberdeen, AB24 3UE, Scotland, UK; E-Mails: j.tabudravu@abdn.ac.uk (J.T.); w.houssen@abdn.ac.uk (W.H.); h.deng@abdn.ac.uk (H.D.); 4 Department of Pharmacognosy, Faculty of Pharmacy, Mansoura University, Mansoura 35516, Egypt

**Keywords:** micromonosporaceae, actinomycete, pyrazinone, mangrove

## Abstract

We report the structural characterization of a new pyrazinone analogue; butrepyrazinone, which was isolated from a new actinomycete strain *Verrucosispora* sp. K51G recovered from Ghanaian mangrove river sediment. Spectroscopy-guided fractionation led to the isolation of a compound from the fermentation culture and a combination of NMR spectroscopy, high-resolution mass spectrometry and computer-aided calculations revealed that butrepyrazinone (**10**) possesses an unusual methylation pattern on the pyrazinone ring. Butrepyrazinone (**10**), however, displayed no antibacterial activity against Gram-positive *S. aureus* ATCC 25923, the Gram-negative *E. coli* ATCC 25922 and a panel of clinical isolates of methicillin-resistant *S. aureus* (MRSA) strains, suggesting that **10** may act as a signal molecule for this strain. Although the same molecule has been synthesized previously, this is the first report to disclose the discovery of butrepyrazinone (**10**) from nature.

## 1. Introduction

**Figure 1:**
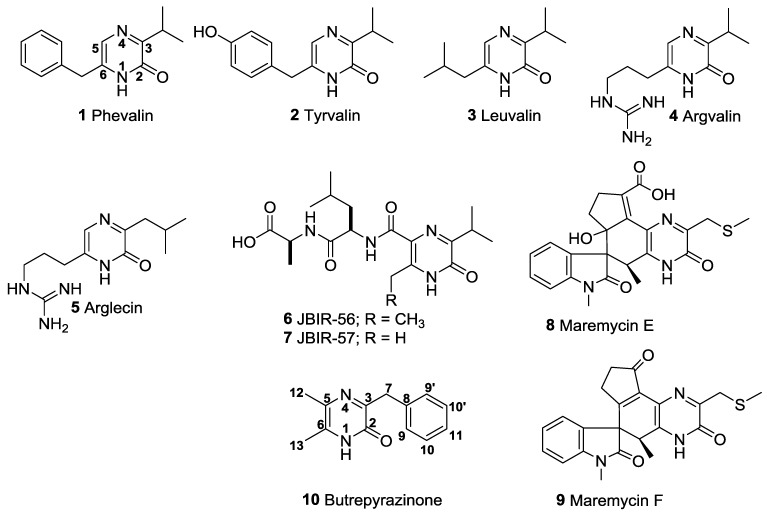
Substitution patterns in pyrazinone compounds isolated from microbes.

The pyrazinone ring is a non-aromatic heterocyclic ring with two nitrogens that are situated para- to each other. Chemically, they are derived from the aromatic pyrazine ring by single oxidation of one of the carbon atoms as opposed to double oxidation, which gives the diketopiperazine ring common in the structures of many drugs in current use. A gradually growing number of microbes have been cited in the literature for their ability to biosynthesize pyrazinone compounds. Examples of microbes from which pyrazinones have been characterized include: Methicillin resistant *Staphylococcus aureus* phevalin (**1**), tyrvalin (**2**) and leuvalin (**3**) [1,2] and *Streptomyces* sp. phevalin (**1**), arglecin (**5**), argvalin (**4**), JBIR-56 (**6**), and JBIR-57 (**7**) [3,4]. It appears that pyrazinones are derived from the multidomain non-ribosomal peptide synthetase (NRPS) assembly line, which involves a reduction in the final step to generate dipeptide aldehyde, followed by cyclization of imine functionality promoted by nucleophilic attack of the aldehyde by the α-amine of one amino acid [3,4]. Hence, the C-5 of the pyrazinone ring is mostly un-substituted (**1**–**5** in Figure 1). While the absence of a substituent in the C-5 of the pyrazinone ring is understandable, based on the current literature available, there exists another group of pyrazinone compounds that possess substituents on both the C-5 and C-6 carbons of the pyrazinone skeleton (Figure 1). For example, JBIR-56 (**6**) and 57 (**7**), maremycin E (**8**) and F (**9**) possess C-5 and C-6 substituents through biosynthetic mechanisms that are not clear [3,4,5].

During our investigations of several *Micromonospora* sp. derived from sediments collected from the Butre River in the western region of Ghana, we came across a *Verrucosispora* sp. K51G, which was found to produce a new pyrazinone butrepyrazinone (**10**) (Figures S1 and S2). The structure of butrepyrazinone (**10**) is typical of what is currently known to characterize the naturally occurring pyrazinone compounds. However, butrepyrazinone (**10**) possesses two methyl substituents at both the C-5 and C-6 positions rendering its biosynthesis difficult to understand completely.

In this communication, we describe the taxonomy and large-scale fermentation of the producing strain and the extraction, isolation, physico-chemical characterization, antimicrobial screening and the structure determination of butrepyrazinone (**10**).

## 2. Results and Discussion

### 2.1. Sediment Sample Collection Sites

The western region of Ghana is particularly noted for large stretches of mangroves that have become characteristically embedded in the life of the natives. An important river, called the Butre, cuts across a large section of these mangroves before entering the sea. We collected sediment samples from the river at four different sites approximately 100 m apart. *Verrucosispora* sp K51G was isolated from one of these sediments (coordinates: 4°49′56.19ʺ N and 1°54′52.00ʺ W).

### 2.2. Taxonomy of Strain Verrucosispora sp. K51G (Genbank Number KM196613)

An almost-complete 16S rDNA gene sequence for strain K51G (1461 nt) was determined and compared with corresponding sequences of all recognized species within the genus *Verrucosispora* [6] and related taxa in the family *Micromonosporaceae* [7,8,9].

**Figure 2:**
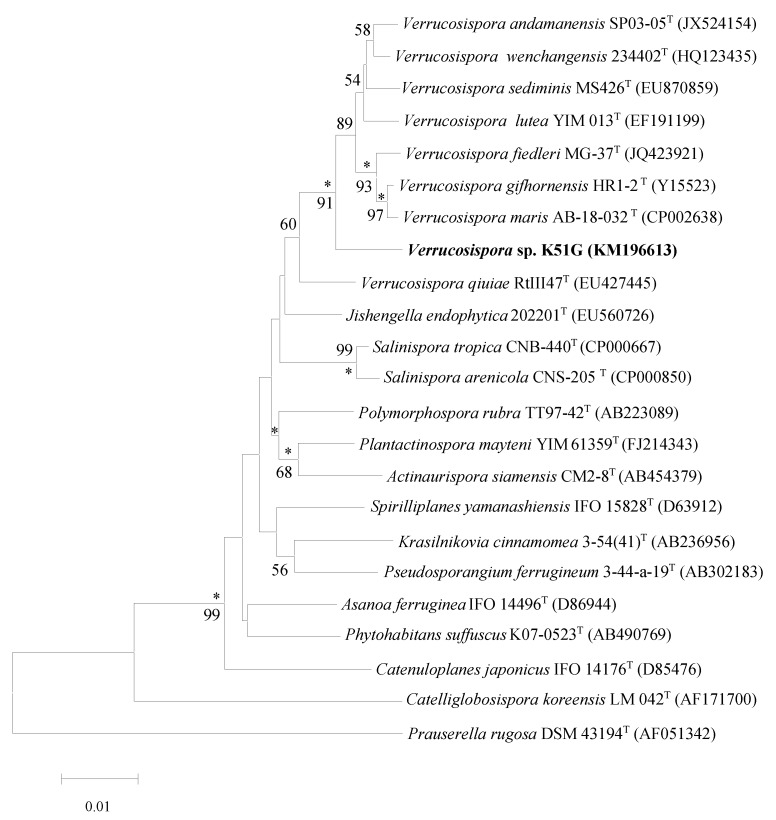
Neighbor-joining tree based on nearly complete 16S rDNA gene sequences (1461 nt) showing relationships between isolate K51G and representatives of genera classified in the family *Micromonosporaceae*. Asterisks indicate branches of the tree that were also found using the maximum-likelihood and maximum-parsimony tree-making algorithms. The numbers at the nodes indicate levels of bootstrap support (%) based on a neighbor-joining analysis of 1000 re-sampled datasets; only values at or above 50% are given. T type strain, Bar 0.01 substitutions per nucleotide position.

The strain showed greater than 98% 16S rDNA gene sequence similarity to the type strains of recognized species of the genus *Verrucosispora*, but in the neighbor-joining phylogenetic tree (Figure 2) based on 16S rDNA gene sequences it formed a distinct phyletic line. The isolate showed the highest level of 16S rDNA gene similarity with the type strain of *Verrucosispora lutea* YIM013^T^ (98.44%, a value that corresponded to 22 nucleotide differences at 1414 locations). Relatively high similarity values were shown with the type strains of *V. wenchangensis* 234402^T^ (98.29%), *V. fiedleri* MG37^T^ (98.28%), *V. gifhornensis* DSM44337^T^ (98.28%), *V. maris* AB18032^T^ (98.22%), *V. sediminis* MS426^T^ (98.20%) and *V.andamanensis* SP03-05^T^ (98.07%). According to the phylogenetic analysis, the strain is clearly distinguished from the other members of the genus *Verrucosispora* and could be representing a new species within the genus *Verrucosispora*.

### 2.3. Structure Determination of Compound 10 (Butrepyrazinone)

A seven-day grown seed culture of *Verrucosispora* sp was used in the inoculation of two L starch casein media in the presence of the Diaion HP-20 resin (50 g/L medium). After 28-day fermentation, the aqueous solution was filtered and HP20 resin was extracted with methanol (3 × 500 mL). The crude extract was concentrated under vacuum and followed by partition using a modified Kupchan method [10]. The purification was achieved through Sephadex LH-20 chromatography (GE Healthcare, Little Chalfont, UK) followed by semi-preparative reversed-phase HPLC resulting in the isolation of **10** (6.2 mg).

Butrepyrazinone was isolated as a colorless amorphous powder soluble in chloroform. The HRESIMS of this compound gave *m/z* = 215.1178 (M + H)^+^ and 237.0998 (M + Na)^+^ indicating a molecular formula of C_13_H_14_ON_2_ (Δ = +0.030 ppm) with 8 degrees of un-saturation. Analysis of the ^1^H, ^13^C and multiplicity edited gHSQCAD spectra suggested the presence of 5 quaternary, 5 methine, 1 methylene and 2 methyl carbons. With the aid of the ^1^H-^13^C gHSQCAD data (Table 1) all protons were assigned to their directly bonded carbon atoms. The presence of three aromatic protons at δ_H_ 7.40 (2H, d, *J* = 6 Hz, H-9/9′), 7.25 (2H, t, *J* = 6 Hz, H-10/10′) and 7.18 (1H, t, *J* = 6 Hz, H-11) suggested a mono-substituted benzene ring with the proton at δ_H_ 4.10 (2H, s, H-7) strongly indicating a methylene benzylic position borne between two rings. The corresponding δ_C_ resonances for this mono-substituted benzene ring and attached methylene benzylic moiety were observed at δ_C_ 129.9 C-9/9′, 128.8 C-10/10′, 126.9 C-11 and 39.6 C-7 respectively. Further confirmation of the first sub-structure obtained from the 1D NMR analysis was placed on ^1^H-^1^H gCOSY data for H-7/H-9,9′, H-9,9′/H-7, H-10,10′, H-10,10′/H-9,9′ and H-11/H-9,9′ reinforced by gHMBCAD peaks from C-7 to H-9,9′, C-9,9′ to H-7, H-10,10′, H-11, C-10,10′ to H-9,9′ and C-11 to H-9,9′. Another pronounced feature of the ^1^H NMR spectrum of butrepyrazinone is the presence of the methyl protons δ_H_ 2.28 (3H, s, H-13) and δ_H_ 2.21 (3H, s, H-12) which by their chemical shifts and multiplicity were indicative of methyl groups situated on quaternary sp^2^ hybridized carbon atoms. Using the δ_C_ data 157.5 C-2, 154.6 C-3, 132.2 C-5 and 129.9 C-6 the pyrazinone ring with two methyl substituent was constructed based on careful examinations of chemical shifts, unsaturation number of 4, and gHMBCAD data correlations C-2 to H-7, C-3 to H-7, H-12, C-5 to H-12, H-13 and C-6 to H-13, H-12. Furthermore, a gCOSY correlation from H-7/H-12 put the placement of the methyl groups on C-5 and C-6 beyond all reasonable doubt. All NMR data obtained for this compound are summarized in Table 1.

**Table 1:** ^1^H and ^13^C NMR data of butrepyrazinone in CDCl_3_. δ in ppm, *J* in Hz.

Position	δ_H_ Mult (*J* Hz)	δ_C_ Mult	HMBC
NH-1			
2		157.5, C	7
3		154.6, C	7, 12
N-4			
5		132.2, C	12, 13
6		129.9, C	13, 12
7	4.10, s	39.6, CH_2_	9, 9′
8		138.3, C	7, 10, 10′
9/9′	7.40, d (6)	129.9, (CH)_2_	7, 10, 10′, 11
10/10′	7.25, t (6)	128.8, (CH)_2_	9, 9′
11	7.18, t (6)	126.9, CH	9, 9′
12	2.21, s	16.7, CH_3_	
13	2.28, s	18.9, CH_3_	

**Figure 3:**
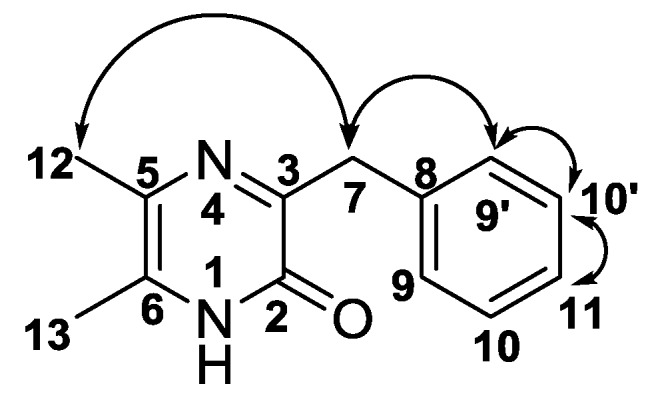
Selected COSY data for butrepyrazinone.

**Figure 4:**
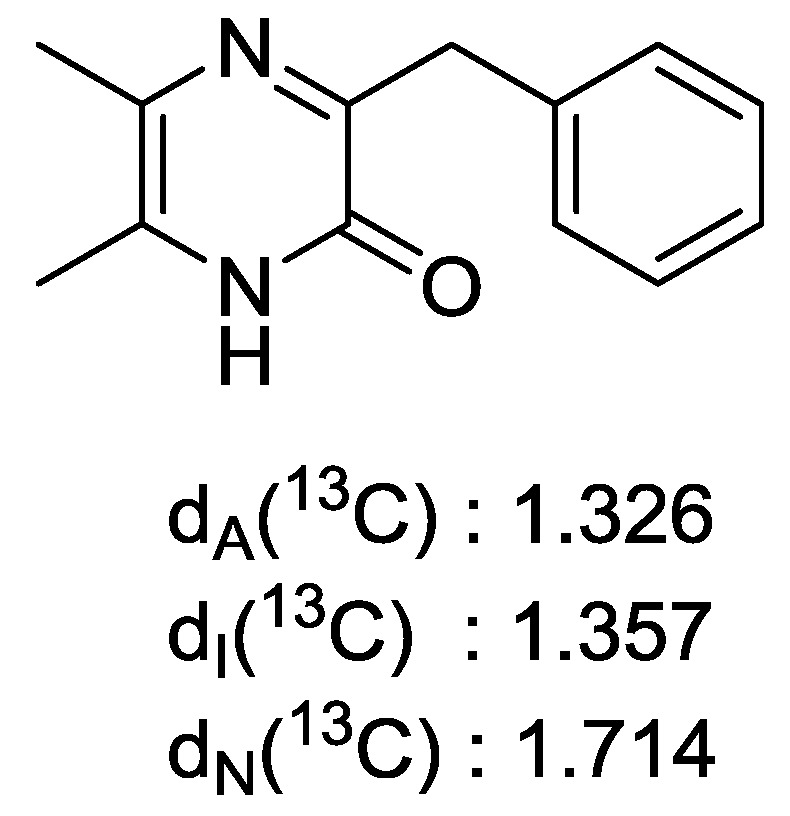
The best candidate structure calculated by the Structure Elucidator with ^13^C chemical shift deviations between experimental and predicted of the HOSE-code (d_A_) Incremental Method (d_I_) and Artificial Neural Net (d_N_).

Further confirmation of the structure was performed by calculating all possible structures using the ACD/LABS Structure Elucidator [11] software as follows: The proton NMR and 2D NMR data including Edited-gHSQCAD, gCOSY, gHMBCAD and ROESY, the molecular formula plus a predetermined mono-substituted benzene ring were entered into the software. Non-standard correlations [12] were detected notably in the gCOSY correlation between H-7 and H-12, and in the HMBC correlations between C-3 and H-12, and C-8 and H-12. These were automatically resolved by the software and calculations resulted in 14 possible structures (Figures S8) of which the proposed structure was placed as the best possible candidate (Figure 3 and Figure 4). The low number of the chemical shift deviations of the HOSE-code (d_A_) [13], Incremental Method (d_I_) [14], and Artificial Neural Net (d_N_) [14] ^13^C chemical shifts indicate that the proposed structure is correct.

**Scheme 1:**
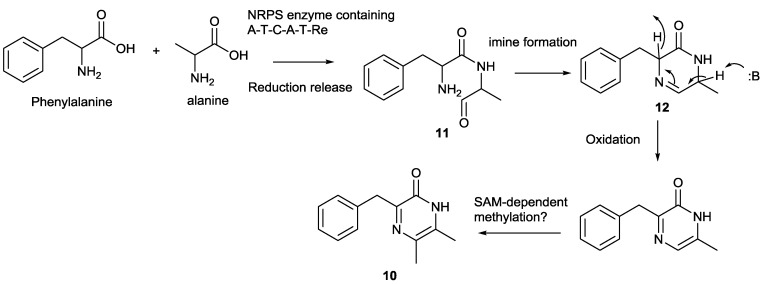
Proposed biosynthetic pathway of butrepyrazinone from *Verrucosispora* sp. K51G. NRPS: Non-ribosomal peptide synthetase; A: Adenylation domain; T: Thiolation domain; C: Condensation domain; Re: NADH-dependent reduction domain.

### 2.4. Proposed Biosynthetic Pathway for Butrepyrazinone

Based on the chemical logic of other pyrazinones in the literature [1,3], we proposed the biosynthesis of butrepyrazinone as shown in Scheme 1. A multidomain non-ribosomal peptide synthetase should be involved in the biosynthesis of butrepyrazinone, and the assembly line is likely to use one phenylalanine and one alanine unit with the reduction reaction in the final step to generate the dipeptide aldehyde (**11**), which is cyclized to afford the imine intermediate (**12**) (Scheme 1). The imine intermediate is further oxidized and methylated to generate butrepyrazinone. The last two steps could result from two different enzymes, oxidase and SAM-dependent methyl transferase. However, one should not exclude the possibility of one hybrid enzyme with two functional domains. This can be exemplified from a recent report of the identification of an epoxidase C-methyltranferase bifunctional fusion protein PsoF in the biosynthetic pathway of Aspergillal polyketide natural products Pseurotins [15]. *In vitro* studies demonstrated that PsoF, a single polypeptide containing a methyl transferase (MT) and a FAD-dependent monooxygenase (FMO), can act as a trans C-methylation of the backbone of the growing polyketide chain and catalyze the stereospecific epoxidation in the late stage of the biosynthesis [15].

### 2.5. Antibacterial Activity of Butrepyrazinone

Butrepyrazinone was tested for its antibacterial activity against the Gram-positive *S. aureus* ATCC 25923, the Gram-negative *E. coli* ATCC 25922 and a panel of clinical isolates of methicillin-resistant *S. aureus* (MRSA) strains (Table 2). The compound, up to 25 μg/mL, did not show antibacterial activity against any strain while rifampicin was distinctly active with MIC values (0.001–0.01 μg mL^−1^).

**Table 2:** Strains used in antibacterial screening of butrepyrazinone.

Strain	Source of Isolate
*S. aureus* ATCC 25923 ^a^	ATCC
*E. coli* ATCC 25922 ^a^	ATCC
SMRSA 105	Toe wound
SMRSA 124	Open wound
SMRSA 116	Knee abscess
EMRSA 15	Urine infection

^a^ Laboratory strains.

All clinical isolates were obtained from the NHS Grampian Microbiology Diagnostic Laboratory, Aberdeen Royal infirmary.

Abbreviations: EMSRA = epidemic MRSA, SMRA = Scottish MRSA.

## 3. Experimental Section

### 3.1. General Experimental Procedures

NMR data were acquired on a Varian VNMRS spectrometer operating at 600 MHz for proton and 150 MHz for carbon. High-resolution mass spectrometric data were obtained using a Thermo Instruments MS system (LTQ XL/LTQ Orbitrap Discovery, Thermo Scientific, Bremen, Germany) coupled to a Thermo Instruments HPLC system (Accela PDA detector, Accela PDA autosampler and Accela pump, Thermo Scientific, Bremen, Germany). The following conditions were used: Capillary voltage 45 V, capillary temperature 260 °C, auxiliary gas flow rate 10–20 arbitrary units, sheath gas flow rate 40–50 arbitrary units, spray voltage 4.5 kV, mass range 100–2000 am (maximum resolution 30,000). HPLC separations were carried out using a Phenomenex Luna reverse-phase (C18 250 × 10 mm, L × i.d., Phenomenex, Macclesfield, UK) column connected to a Waters 1525 Binary HPLC pump Chromatograph (Waters, Milford, MA, USA) with a 2998 PDA detector (Waters, Milford, MA, USA), column heater and in-line degasser. Detection was achieved on-line through a scan of wavelengths from 200 to 400 nm. Diaion HP-20 was obtained from Resindion S.R.L., a subsidiary of Mitsubishi Chemical Co., Binasco, Italy. All solvents used throughout were HPLC-grade and purchased from Sigma-Aldrich (Taufkirchen, Germany) through a Ghana-based agent (Huge Limited, Accra, Ghana). Sephadex LH-20 (25–100 μm) was purchased from GE Healthcare (Little Chalfont, UK). TLC Silica gel plates (60 F_254 nm_) were purchased from Merck KGaA (Darmstadt, Germany).

### 3.2. Identification of Verrucosispora sp K51G

Genomic DNA of strain K51G was purified with a DNA extraction kit (Zymo Research, Irvine, CA, USA). PCR amplification of the 16S rDNA gene was performed with Go Taq Hot Start master mix (Promega Corp., Madison, WI, USA) according to the manufacturer’s instructions. The almost complete (1461 bp) 16S rDNA gene sequence of strain K51G was determined by using ABI PRISM 3730 XL automatic sequencer (Applied Biosystems, Foster City, CA, USA). Pairwise levels of similarity of the nearly complete 16S rRNA gene sequence of strain K51G were determined on the EzTaxon-e Server (Chunlab Inc., Seoul, South Korea) by using identity analysis [16]. Multiple alignments with sequences from closely related species were performed by using the program CLUSTAL W in the MEGA6 software package [17]. Phylogenetic tree was constructed with the neighbor joining (NJ), maximum-likelihood (ML), maximum-parsimony (MP) algorithms in [18,19,20]. Evolutionary distances were calculated using model of Jukes and Cantor (1969) [21]. Topologies of the resultant trees were evaluated by bootstrap analysis [22] based on 1000 re-samplings.

### 3.3. Isolation of Verrucosispora sp K51G and Preliminary Screening of the Secondary Metabolites

Sediment sample was collected from the Butre River in the western region of Ghana (coordinates: 4°49′56.19ʺ N and 1°54′52.00ʺ W). *Verrucosispora* sp. K51G was isolated from the swampy mangrove sediment using the procedure outline as follows. A small portion of the sediment was transferred into sterile 50 mL tubes, covered and the cap wrapped tightly with cellophane to prevent the entry of water or steam. The tube containing sample was then immersed in a water bath set at temperature 55 °C and heated for 3 h to eliminate non-sporulating bacteria. The sample was then suspended in 10 mL of sterile water under a clean bench and filtered using a previously autoclaved filter paper. The resultant filtrate was serially diluted to 10^−1^, 10^−2^ and 10^−3^ of the original volume. Subsequently, 50 μL of each of the three dilutions were transferred to previously prepared casein starch agar plates (agar, 15 g; soluble starch, 10 g; dibasic potassium phosphate, 2 g; potassium nitrate, 2 g; sodium chloride, 2 g; casein, 0.3 g; magnesium sulfate heptahydrate, 0.05 g; calcium carbonate, 0.02 g; iron sulfate heptahydrate, 0.01 g; sea salt, 33 g and 1 L of tap water) supplemented with nalidixic acid and nystatin at 2.5 μg/L each. This medium is highly recommended for the detection and subsequent culture for saccharolytic marine bacteria and actinomycetes. Different colonies of *Micromonospora* sp. were observed after three weeks of incubation at 28 °C. Subsequently, each colony was repeatedly transferred and streaked on different fresh casein starch plates with the aid of autoclaved tooth picks until pure strains were obtained. One individual bacteria colony from strain *Verrucosispora* sp. K51G was fermented in 50 mL of starch casein liquid media using previously autoclaved 250 mL Erlenmeyer flasks plugged with non-absorbent cotton wool. The culture was allowed to grow for ten days at 28 °C with continues agitation at 200 rpm. Diaion HP-20 (50 g/L) was added to the culture three days prior to harvesting. The culture was then filtered under suction through a piece of glass wool placed in a Buchner funnel and the filtrate was discarded. The Diaion HP-20 resins were then soaked repeatedly and alternatively in CH_3_OH and CH_2_Cl_2_ and the resultant extracts were combined and dried under vacuum to give the crude extract. The extract was then subjected to HPLC/HRESIMS analysis. Analysis of the resultant data from HPLC/HRESIMS revealed three minor and one major peak for m*/z* indicating the presence of butrepyrazinone. This mass was entered as a query in the Natural Products Identifier AntiBase 2013 software (Wiley, Chichester, West Sussex, UK) to check whether this molecule was new or novel. The search came with no relevant hits and the *Verrucosispora* sp. K51G was tagged as one of the interesting strains for further investigation.

### 3.4. Large Scale Fermentation of Verrucosispora sp. K51G

A pure colony of *Verrucosispora* sp. K51G was used to inoculate a 250 mL Erlenmeyer flask containing 50 mL of liquid starch casein media as described above but without agar. After seven days of incubation at 28 °C with continuous agitation at 200 rpm, the culture was used to inoculate two 2 L conical flasks containing already autoclaved 1 L starch casein liquid media and plugged with non-absorbent cotton wool. The two 2 L flasks were incubated at 28 °C with continuous agitation at 250 rpm for three weeks. Diaion HP-20 resin (50 g/L) was added under sterile conditions using a serological pipette to both flasks after three weeks and returned back to the incubator set at 28 °C for another one week of continuous agitation at 250 rpm. After four weeks of incubation, the two 1 L cultures were harvested and filtered under pressure using a piece of glass wool placed in a Buchner funnel. The filtrate was discarded and the residue consisting mainly of the Diaion HP-20 resin with adsorbed organics was repeatedly and alternatively extracted with CH_3_OH and CH_2_Cl_2_. The CH_3_OH and CH_2_Cl_2_ extracts were combined and concentrated under reduced pressure to give 768 mg of a light yellow total crude extract (TCE).

### 3.5. Extraction, Isolation and Purification of Compound

The TCE was suspended in 150 mL of H_2_O and extracted three times with the same volume of CH_2_Cl_2_.

The CH_2_Cl_2_ layer (425 mg) was dried under vacuum and suspended in a 200 mL (9:1 v/v) mixture of methanol and water. This mixture was then placed in a 1 L separating funnel and extracted three times with the same volume of hexane. The 9:1 v/v mixture of methanol and water was phase adjusted to 5:5 v/v methanol and water by adding 160 mL of water to the 9:1 v/v methanol water mixture. The 5:5 v/v methanol water mixture was then extracted three times with the same volume of CH_2_Cl_2_. The CH_2_Cl_2_ layer was then dried under vacuum to give 130 mg of extract. This extract was subsequently loaded on a gravity column packed with Sephadex LH-20 (GE Healthcare Little Chalfont, UK) and eluted with a 5:5 v/v mixture of CH_3_CN and CH_3_OH. Four fractions were collected from the Sephadex LH-20 run and labeled SF1-4. HPLC/HRESIMS showed that SF2 (45 mg) contained the compound of interest. This fraction was therefore subjected to HPLC separation and purification using a Phenomenex Luna C_18_ column (C_18_ 250 × 10 mm, L × i.d., Phenomenex, Macclesfield, UK). Gradients of H_2_O: CH_3_CN (100% H_2_O to 100% CH_3_CN in 30 min and hold for 20 min) were used as eluent with column flow rates set at 1.5 mL/min to afford butrepyrazinone (6.2 mg).

Butrepyrazinone (**10**): Colorless amorphous powder; UV (CH_3_OH), λ_max_: 230, 338 nm.; IR (neat) ν_max_: 2361, 2340, 2330, 1992, 1648, 749 cm^−1^; ^1^H NMR (CDCl_3_, 600 MHz) and ^13^C NMR (CDCl_3_, 150 MHz) data, see Table 1; HRESIMS *m/z* 214.1178 (calcd. for C_13_H_14_ON_2_, 214.1178).

### 3.6. Antibacterial Activity of Butrepyrazinone

The antibacterial activity of butrepyrazinone was evaluated against *S. aureus* ATCC 25923, *E. coli* ATCC 25922 and a panel of methicillin-resistant *S. aureus* clinical isolates obtained from the NHS Grampian Microbiology Diagnostic Laboratory using slight modifications of the previously described method [23]. In brief, bacterial strains were grown in Müller-Hinton (MH) broth [24] to early stationary phase and then diluted to an OD_620_ = 0.1. The assays were performed in a 96-well micro titer plate format. Rifampicin and butrepyrazinone were dissolved in DMSO (Sigma, Taufkirchen, Germany), and the effect of different dilutions in broth on the growth was assessed after 18 h incubation at 37 °C using a Labsystems iEMS Reader MF plate reader (MTX Lab System, Vienna, VA, USA) at OD_620_. The MIC was determined as the lowest concentration showing no growth compared to the MH broth control. DMSO up to 10% was shown to have no antibacterial effect.

## 4. Conclusions

Butrepyrazinone was isolated from *Verrucosispora* sp. K51G recovered from a sediment collected from the Butre River, which is situated in the western region mangroves of Ghana. This represents the first report of a pyrazinone compound from a *Verrucosispora* sp. The compound, butrepyrazinone did not show any antibacterial activity against the Gram-positive *S. aureus* ATCC 25923, the Gram-negative *E. coli* ATCC 25922 and a panel of clinical isolates of methicillin-resistant *S. aureus* strains and rightfully so. This lack of activity is fully in-line with the current experimental data that suggests that, these compounds do not have detectable antibacterial activity but seem to play a role in the chemical interaction between *S. aureus,* or other skin microbes, and the host. While the structure of butrepyrazinone has been previously synthesized [25,26,27,28,29] it is very interesting to realize that this compound is actually a natural product produced by a very rare strain of marine actinomycetes. Under the current circumstance, a study of the biosynthesis and biological function of this compound in the *Verrucosispora* sp is of utmost interest.

## Supplementary Files

Supplementary File 1
