# Supplementary material for: Butrepyrazinone, a New Pyrazinone with an Unusual Methylation Pattern from a Ghanaian Verrucosispora sp. K51G
Source: Mar Drugs. 2014 Oct 16;12(10):5197–208. doi: 10.3390/md12105197 (PMC4210894; doi:10.3390/md12105197)

Supplementary Information

Figure S1. *Verrucosispora* sp. K51G growing in starch casein liquid media with sea salts.

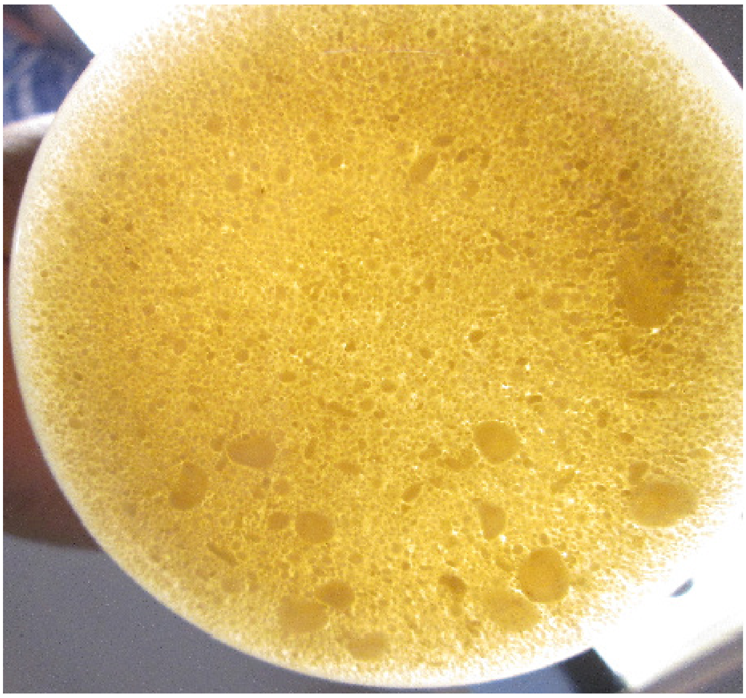

Figure S2. PDA/UV profiles obtained from the HPLC/HRESIMS data of the crude extracts of *Verrucosispora* sp. K51G.

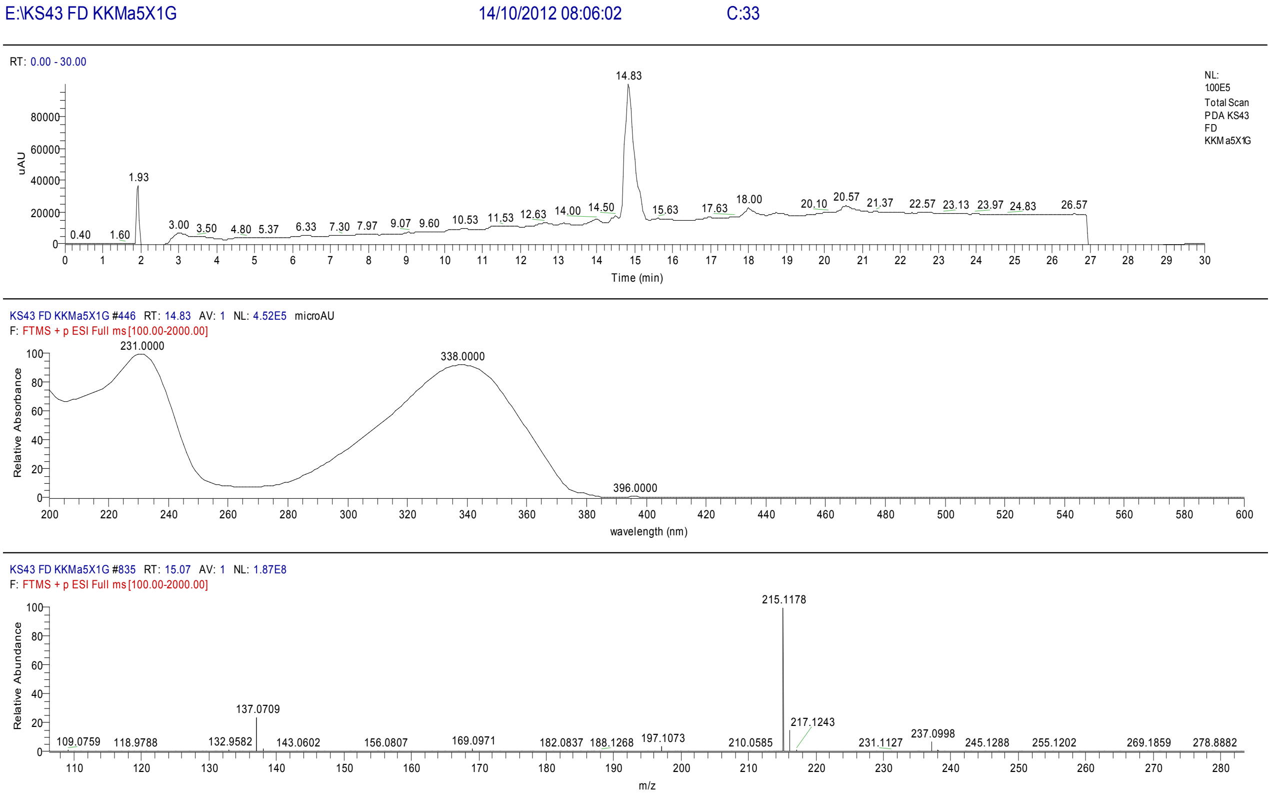

Figure S3. ^{1}H NMR spectrum of butrepyrazinone (**10**).

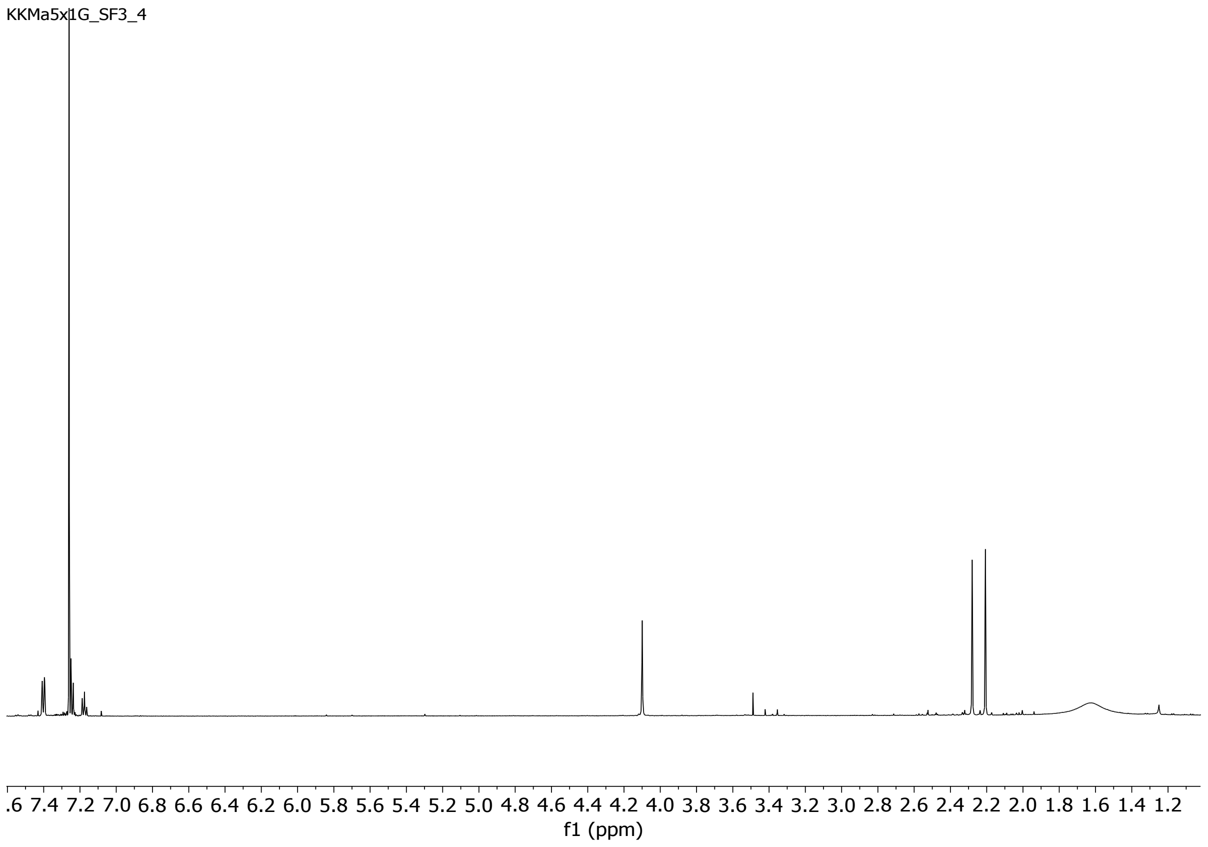

Figure S4. ^{13}C NMR spectrum of butrepyrazinone (**10**).

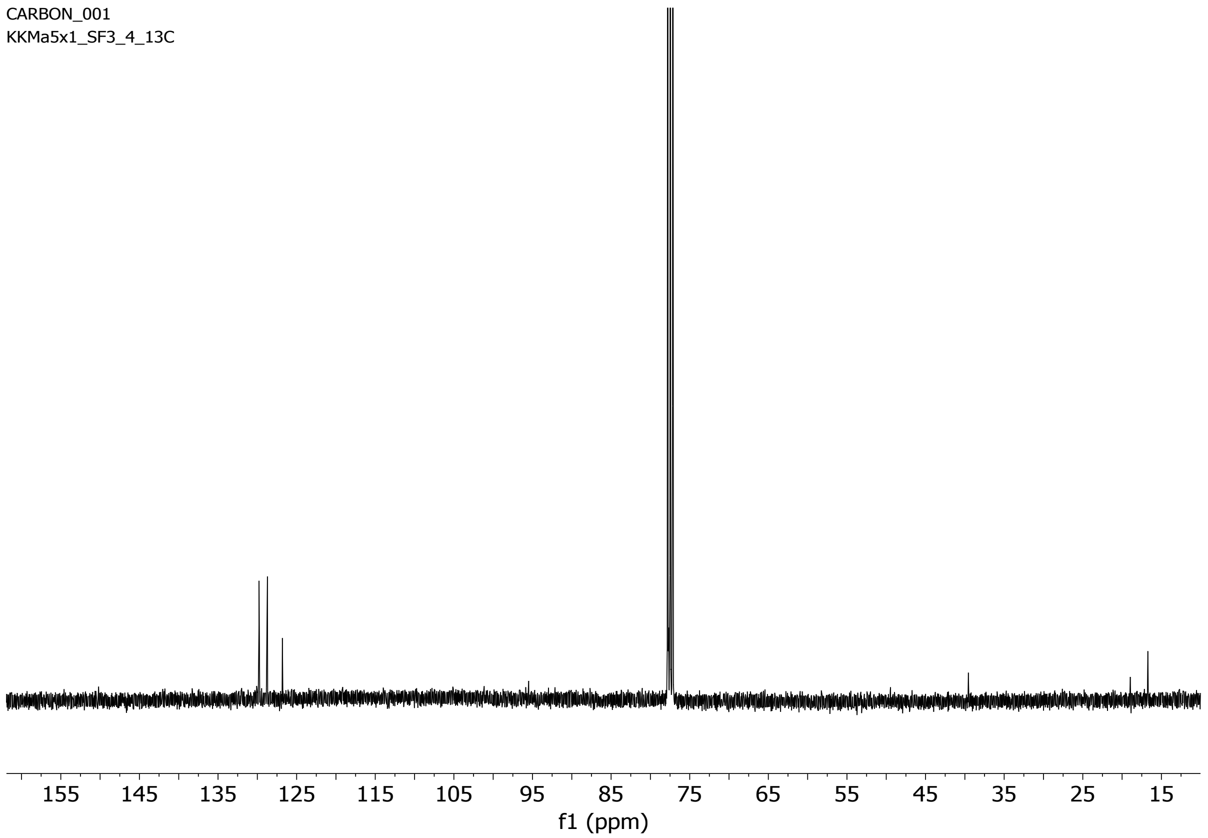

Figure S5. gHSQCAD spectrum of butrepyrazinone (**10**).

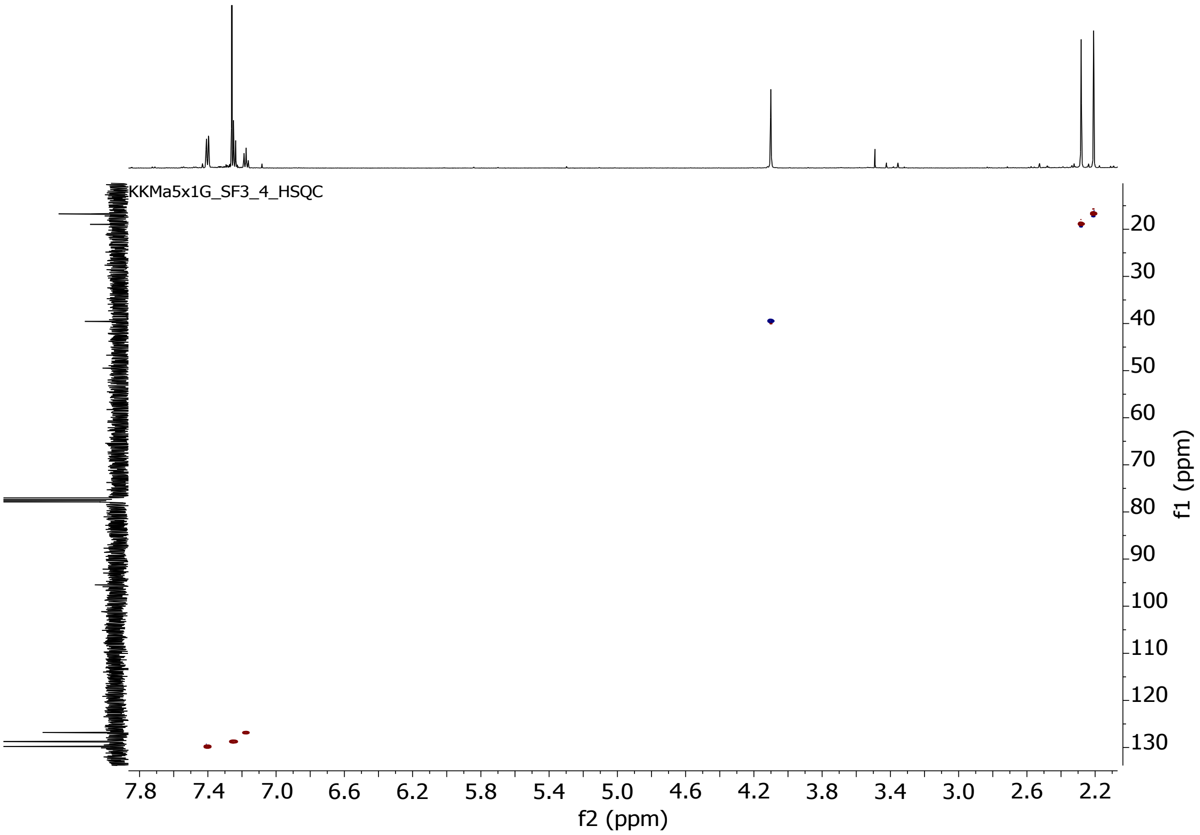

Figure S6. ^{1}H-^{1}H gCOSY spectrum of butrepyrazinone (**10**).

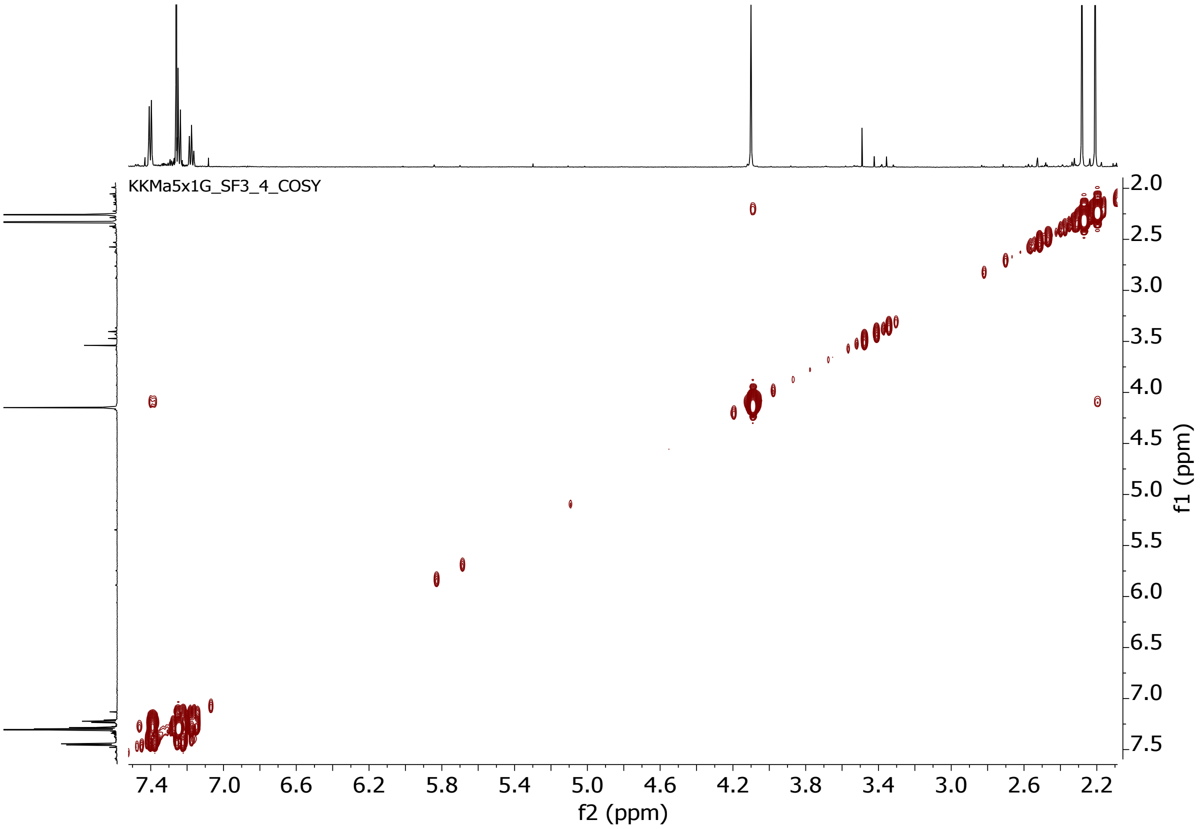

Figure S7. HMBCAD spectrum of butrepyrazinone (**10**).

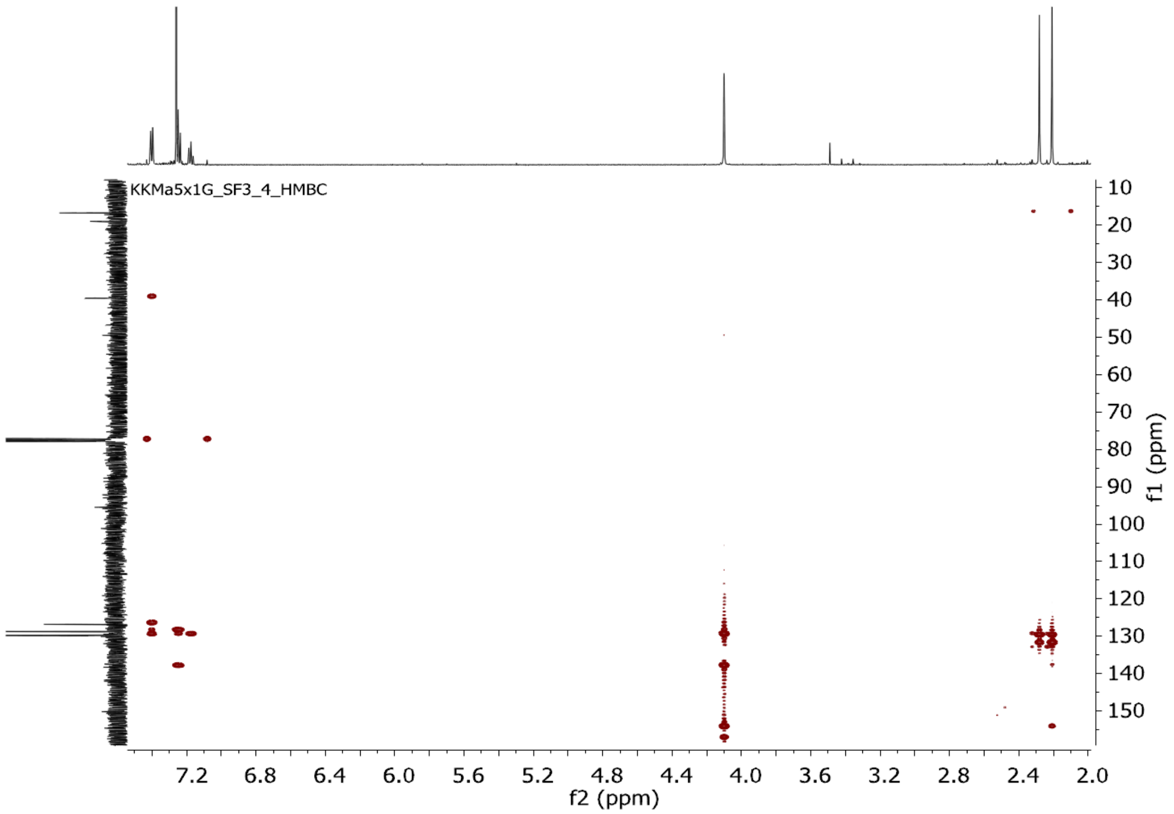

Figure S8. All possible structures calculated by the ACDLabs Structure Elucidator software showing the best candidate in position number 1.

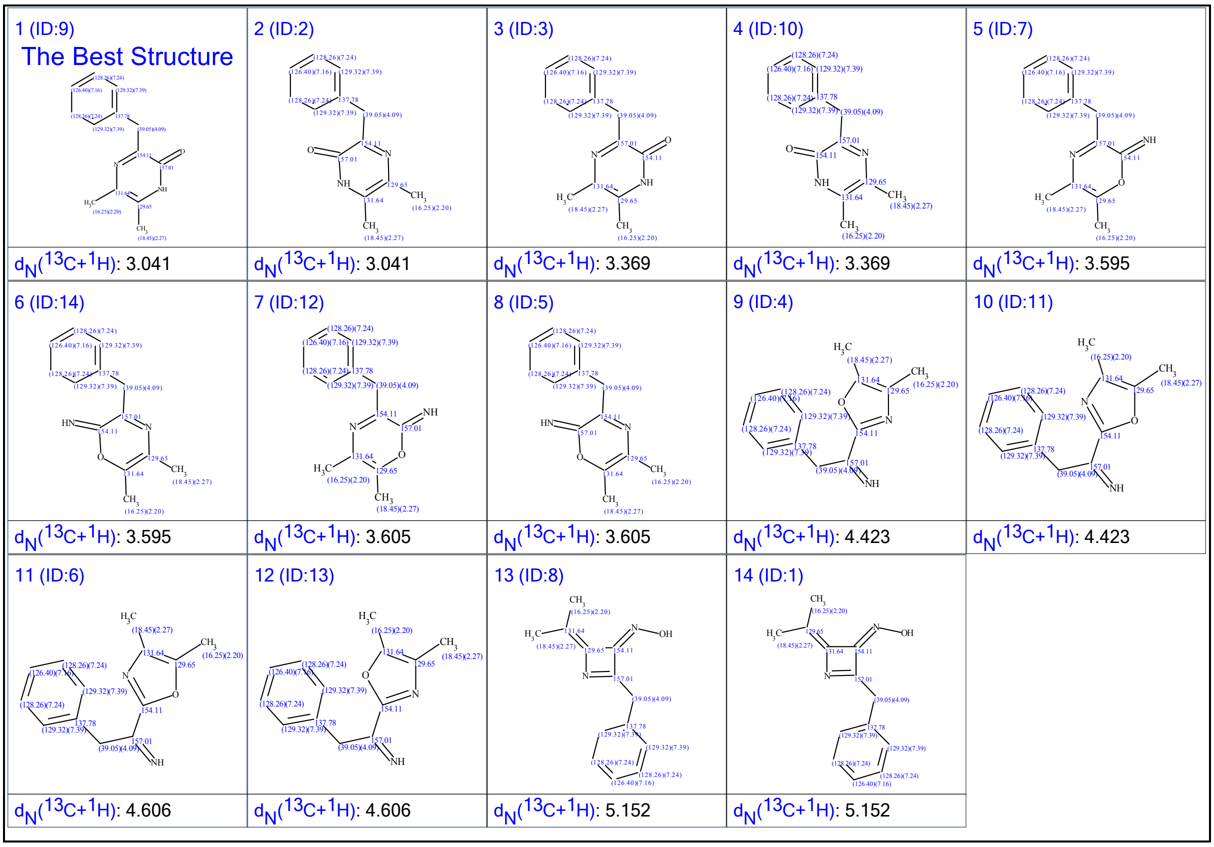

Supplement: Supplementary File 1 [file marinedrugs-12-05197-s001.pdf]
